# A radiochemical lab-on-a-chip paired with computer vision to unlock the crystallization kinetics of (Ba,Ra)SO_4_

**DOI:** 10.1038/s41598-024-59888-6

**Published:** 2024-04-25

**Authors:** Jenna Poonoosamy, Alexander Kaspor, Christian Schreinemachers, Dirk Bosbach, Oskar Cheong, Piotr M. Kowalski, Abdulmonem Obaied

**Affiliations:** 1 Institute of Energy and Climate Research, Forschungszentrum Jülich GmbH, IEK-6): Nuclear Waste Management, 52425 Jülich, Germany; 2 Institute of Energy and Climate Research (IEK-13): Theory and Computation of Energy Materials, Forschungszentrum Jülich GmbH, 52425 Jülich, Germany; 3 JARA Energy and Center for Simulation and Data Science (CSD), 52425 Jülich, Germany; 4 Chair of Theory and Computation of Energy Materials, Faculty of Georesources and Materials Engineering, RWTH Aachen University, Intzestrasse 5, 52072 Aachen, Germany

**Keywords:** Computer vision, Ra-bearing barite, Microfluidics, Crystal growth, Solid solutions, Geochemistry, Mineralogy, Nuclear chemistry

## Abstract

(Ra,Ba)SO_4_ solid solutions are commonly encountered as problematic scales in subsurface energy-related applications, e.g., geothermal systems, hydraulic fracturing, conventional oil and gas, etc. Despite its relevance, its crystallization kinetics were never determined because of radium (226), high radioactivity (3.7 × 10^10^ Bq g^−1^), and utilization in contemporary research, therefore constrained to trace amounts (< 10^−8^ M) with the composition of Ba_*x*_Ra_1-*x*_SO_4_ commonly restricted to *x* > 0.99. What if lab-on-a-chip technology could create new opportunities, enabling the study of highly radioactive radium beyond traces to access new information? In this work, we developed a lab-on-a-chip experiment paired with computer vision to evaluate the crystal growth rate of (Ba,Ra)SO_4_ solid solutions. The computer vision algorithm enhances experimental throughput, yielding robust statistical insights and further advancing the efficiency of such experiments. The 3D analysis results of the precipitated crystals using confocal Raman spectroscopy suggested that {210} faces grew twice as fast as {001} faces, mirroring a common observation reported for pure barite. The crystal growth rate of (Ba_0.5_Ra_0.5_)SO_4_ follows a second-order reaction with a kinetic constant equal to (1.23 ± 0.09) × 10^−10^ mol m^−2^ s^−1^.

## Introduction

Radium (Ra), with isotopes ^223^Ra, ^224^Ra, ^226^Ra, and ^228^ Ra, is a naturally occurring radioactive material (NORM) that results from the radioactive decay of ^238^U and ^232^Th^1^. ^226^Ra is the most relevant isotope explained by its half-life of 1600 years. Due to its strong affinity for barite (BaSO_4_), (Ba,Ra)SO_4_ solid solutions are commonly encountered as problematic scales in various subsurface energy-related applications^2–8^. For instance, wastewater from the extraction and production of conventional oil and gas^9^, hydraulic fracturing^10^ as well as geothermal brines^11^, contain radium. Besides its accumulation in uranium mill tailings^12–16^, ^226^Ra, will play a major role in the safety assessments for nuclear waste repositories^17^. Under certain repository configurations, Ba and Ra, generated from the decay of cesium (fission product) and uranium, respectively, may react with sulfate-rich pore water from the host rock, resulting in the formation of (Ba,Ra)SO_4_ solid solutions^18,19^. Determining the thermodynamic and kinetic parameters that describe the crystallization of (Ba,Ra)SO_4_ is therefore essential for optimizing waste water treatment, site remediation measures, or generating reliable predictions of hydrogeochemical processes in subsurface energy-related applications^7,20^.

In the past 10 years, there has been tremendous effort to investigate the thermodynamic properties and mixing behavior of (Ba,Ra)SO_4_ solid solutions over a wide range of temperatures (23 °C to 90 °C). The summary of these studies, combining batch experiments^21–23^, advanced analytical techniques e.g. time-of-flight secondary ion mass spectrometry (ToF–SIMS) or transmission electron microscopy (TEM)^24–26^ and thermodynamic modelling^27,28^ suggested that the (Ba,Ra)SO_4_ solid solution series is non ideal and is described by a regular mixing model with an interaction parameter of 2.5 ± 0.2 kJ mol^−1^^29,30^. In the case of nuclear waste repositories, it is important to note that supersaturated solutions may exist metastably in the pore water of rock matrices in the subsurface. As a result, it is likely that parameters pertaining to nucleation and precipitation kinetics will play a major role in Ra migration through the repository near-field, making thermodynamic equilibrium calculations insufficient for a reliable prediction of ^226^Ra mobility^18^. Kinetics studies so far have focused on radium uptake into barite and recrystallization processes^31–33^ and for the determination of partition coefficients^34^. To the best of our knowledge, no studies have actually quantified the precipitation kinetics of (Ba_*x*_,Ra_1-*x*_)SO_4_ as a function of solution supersaturation.

One possible explanation would be that conventional methods, e.g., batch crystallizers^35^, to study nucleation and precipitation kinetics would require a significant amount of radium. Experiments involving radium are difficult because of its high specific activity (3.7 × 10^10^ Bq g^−1^)—^226^Ra decays to form gaseous ^222^Rn and a series of other short-lived α-emitting isotopes^36^. Indeed, beside the classical work of Marie Curie (1911)^37^, Doerner and Hoskins (1925)^38^, Hahn (1926)^39^ and Goldschmidt (1940)^40^ on radium, in today’s research radium is usually used at trace concentrations (< 10^–8^ M), or barium is employed as a surrogate^41^.

Microfluidics-based screening and lab-on-a-chip, employed for decades in biotechnology, are now emerging as versatile tools in geosciences for studying fluid-rock interactions^42–44^. Microfluidics, or lab-on-a-chip, is an experiment that is conducted in a microreactor, monitored by time-lapse microscopy, and recently combined with in-operando spectroscopic or synchrontron techniques to monitor phase transformation^45,46^. Such systems can mimic the pore architecture of rocks and allow live monitoring of mineral nucleation, precipitation, and dissolution under well-controlled conditions (temperature and pressure). The methodology was successfully employed to investigate the nucleation of carbonates, sulfates and iron hydroxides in confinement^47–52^, mineral precipitation and dissolution processes^53–57^, or the complex interplay between crystallization and transport that leads to oscillatory zoning phenomena during solid solution precipitation^20^. It is used in radiopharmaceutics for the synthesis of radioactive position emission tomography (PET) tracers^58–60^. Microfluidics requires small quantities of fluid to conduct hundreds of experiments simultaneously. This technology has the potential to revolutionize radio-geochemistry by allowing researchers to work with high concentrations of radium or other radionuclides in solution at low radionuclide inventories. This would keep radiation doses below those of traditional batch experiments. However, the quantity of data resulting from these microfluidic experiments can be quite significant given the robust and high throughput nature of these setups. In addition, the results are mainly 2D optical images, which require significant time and human resources for evaluation, e.g., for the determination of crystal growth rates. To fully leverage the potential of these kinds of experiments, the evaluation processes along with several other bottlenecks must be removed by employing an artificial intelligence (AI) computer vision (CV) methodology in different steps of the workflow.

The aim of this work was to test the use of lab-on-a-chip experiments monitored by time-resolved microscopy in combination with Raman spectroscopy and the application of CV to investigate the crystal growth rate of (Ba,Ra)SO_4_ solid solutions. Reacting solutions were injected into a microfluidic mixer to precipitate (Ba,Ra)SO_4_, where radium is beyond the trace. The experiments were carefully designed to precipitate Ba_0.5_Ra_0.5_SO_4_ under various saturation conditions. The 3D Raman tomographs of the single crystals suggested that the $\left\{210\right\}$ face grew twice as fast as the {001} face, a common behavior for barite^61–63^. A computer vision pipeline was developed to identify crystal habits and track crystal growth from 2D optical images. Our algorithm also included the evaluation of the Ba_0.5_Ra_0.5_SO_4_ crystals in 3D to extract the volume and surface area of single crystals with time. These enabled the determination of the crystal growth rate of Ba_0.5_Ra_0.5_SO_4_, which follows a second order reaction, similar to barite, with a kinetic constant equal to (1.23 ± 0.09) × 10^–10^ mol m^−2^ s^−1^. The lesson learned from this study marks the first building block towards an automated radio-geochemical lab-on-chip.

## Results and discussion

### Evaluation of the solution chemistry and stoichiometric saturation function

**Figure 1:**
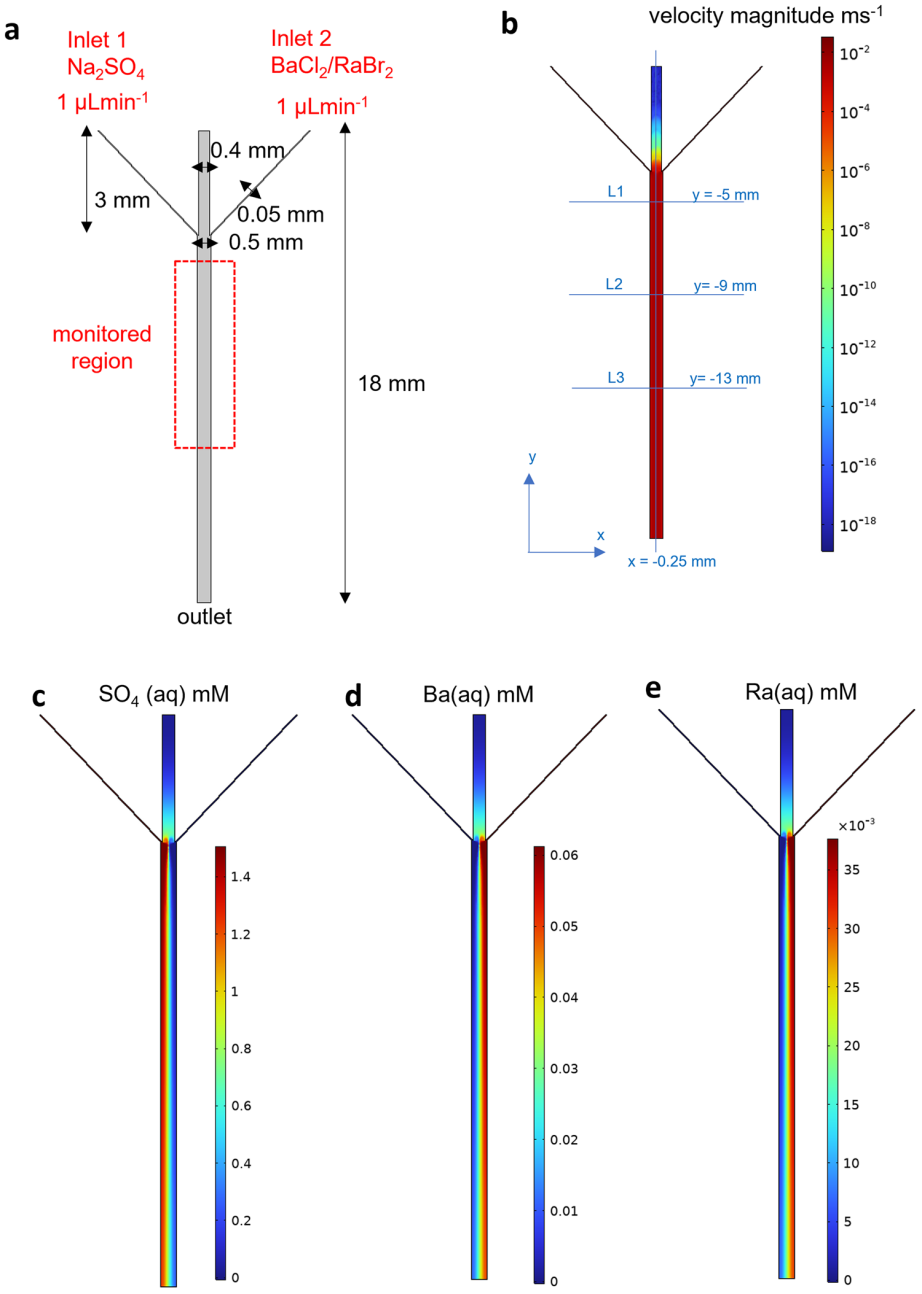
Evaluation of the solution chemistry in the microfluidic reactor for experiment C. (**a**) Design of microfluidic reactor with two inlets where reacting solutions are injected and an outlet. (**b**) Simulated velocity magnitude in the microfluidic reactor with lines 1, 2 and 3 where the solution chemistry is numerically sampled. The line *x* = 0.25 mm marks the middle of the reactor (**c**) sulfate (**d**) barium and (**e**) radium concentrations in the microfluidic reactor at steady state for experiment C.

**Figure 2:**
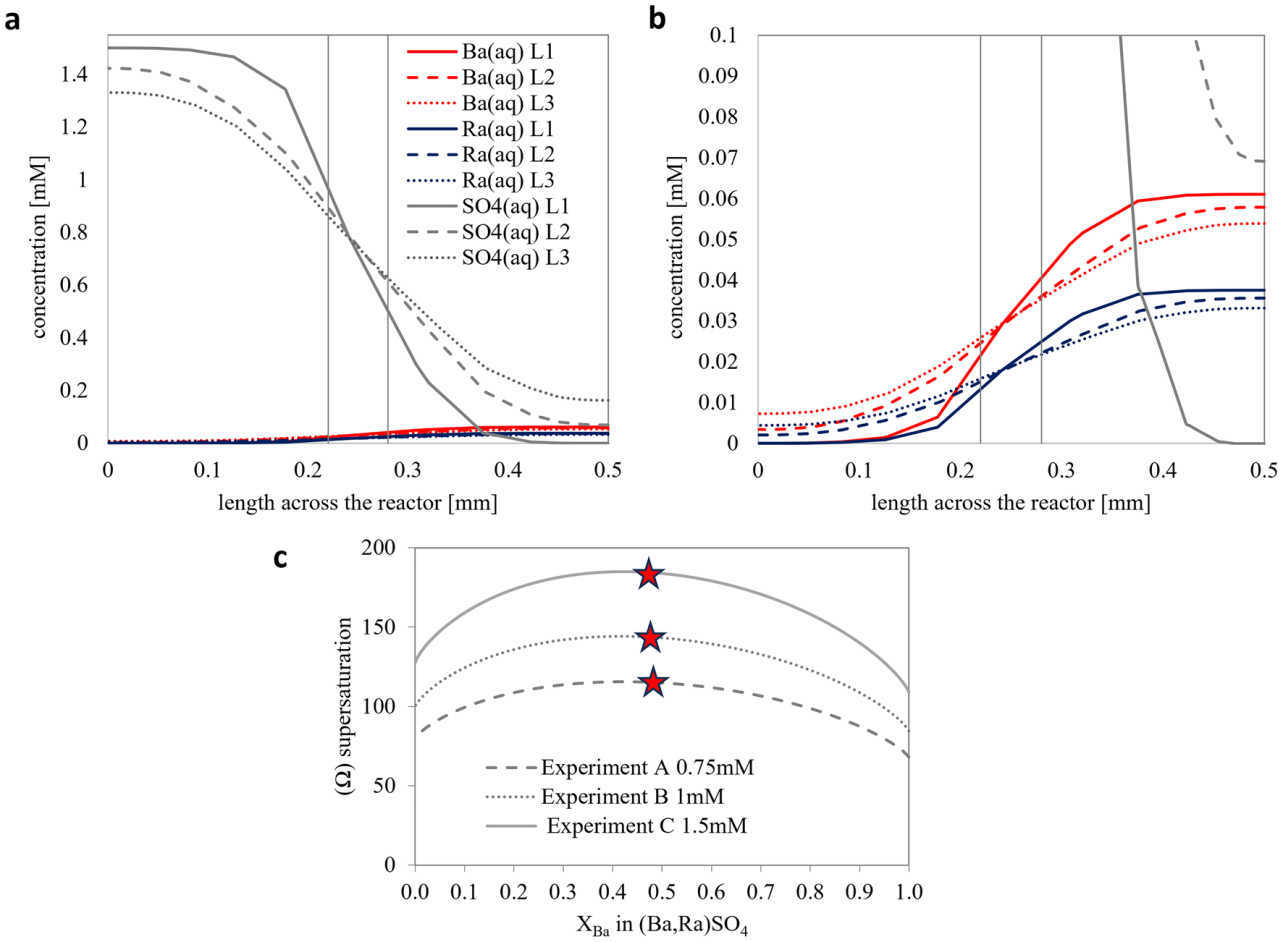
Evaluation of the solution chemistry and supersaturation function Ω_st_ along the mixing zone. (**a**) The concentration of solutes across line 1–3 (c.f. Figure 1) for experiment C with (**b**) a change in the *y*-axis for a better visualization of Ba_(aq)_ and Ra_(aq_ (**c**) The comparison of the Ω_st_ for experiment A, B and C along line 1 at *x* = 0.25 mm with the red stars indicating the thermodynamically most stable solid solution composition based on the aqueous solution chemistry.

The laminar mixing reactor (Fig. 1a) was used to foster the crystallization of (Ba,Ra)SO_4_. Three experiments denoted A, B and C, were conducted using consistent concentrations of barium chloride (BaCl_2_) and radium bromide (RaBr_2_) but with varying concentrations of sodium sulfate (Na_2_SO_4_, 0.75 mM, 1 mM, and 1.5 mM respectively). The fluxes and concentrations of the reacting solutes in the microfluidic channel were calculated using COMSOL Multiphysics^64^ (see details in supplementary note 1). This allowed the evaluation of solute species concentrations at every point in space and time in the microfluidic channel. By coupling this information with geochemical speciation calculations using GEMS^65^, the corresponding saturation indices with respect to the solid solution series were determined. The injection of Na_2_SO_4_ and a mixed solution of RaBr_2_ and BaCl_2_ at a flow rate of 1 µL min^−1^ induces a fluid velocity of about 4 × 10^–3^ m s^−1^ (Fig. 1b). The Reynolds number defined by the ratio of inertial forces to viscous forces is 3.75, indicating a laminar flow whereby the reacting fluids flow in parallel (Fig. 1c–e), without turbulence, and the only mixing that occurs is the result of the diffusion of molecules across the interface (green region of the microfluidic channel in Fig. 1c–e) between the reacting fluids^66^. The concentrations of the reacting solutes for the experiment with 1.5 mM Na_2_SO_4_ across lines 1–3 (Fig. 1b) are plotted in Fig. 2a, b. A concentration gradient develops along the *x*-axis of the reactor, leading to a widening of the mixing zone as the fluids flows toward the outlet. This is evident in Fig. 2a, where a non-zero sulfate concentration is observed across lines 2 and 3 in contrast to line 1.

The solutes react according to Eq. (1) to form (Ba,Ra)SO_4_ solid solutions along the mixing zones.

1 $${x{\text{Ba}}}_{\left(aq\right)}^{2+}+\left(1-x\right){{\text{Ra}}}_{\left(aq\right)}^{2+}+{{\text{SO}}}_{4(aq)}^{2-}\leftrightarrow {{\text{Ba}}}_{x}{{\text{Ra}}}_{(1-x)}{{\text{SO}}}_{4(s)}$$

The stoichiometric supersaturation function ${\Omega }_{st}$ for the solid solutions was computed for the entire compositional range from *X*_Ba_ = 0 to *X*_Ba_ = 1 using the equation below^67^:

2 $${\Omega }_{st}({X}_{{\text{Ba}}})=\frac{{({a}_{{{\text{Ba}}}^{2+}})}^{{X}_{{\text{Ba}}}}{({a}_{{{\text{Ra}}}^{2+}})}^{{X}_{{\text{Ra}}}}({a}_{{{{\text{SO}}}_{4}}^{2-}})}{{\left({K}_{{{\text{BaSO}}}_{4}}{\gamma }_{{{\text{BaSO}}}_{4}}{X}_{{\text{Ba}}}\right)}^{{X}_{{\text{Ba}}}}.{\left({K}_{{{\text{RaSO}}}_{4}}{\gamma }_{{{\text{RaSO}}}_{4}}{X}_{{\text{Ra}}}\right)}^{{X}_{{\text{Ra}}}}}$$

where ${a}_{{{\text{Ba}}}^{2+}}$, ${a}_{{{\text{Ra}}}^{2+}}$ and ${a}_{{{{\text{SO}}}_{4}}^{2-}}$ represent the free ion activities in the aqueous solution considering the extended Debye–Huckel ionic strength activity model; ${K}_{{{\text{BaSO}}}_{4}}$ and ${K}_{{{\text{RaSO}}}_{4}}$, the solubility products of the end-members BaSO_4_ and RaSO_4_ equal to 10^–9.97^ mol^2^ L^−1^ and 10^–10.26^ mol^2^ L^−1^ respectively at 298.15 K (cf. Klinkenberg et al. ^25^); and ${X}_{{\text{Ba}}}$ and ${X}_{{\text{Ra}}}$, the molar fractions of BaSO_4_ and RaSO_4_ in the solid. ${\gamma }_{{{\text{BaSO}}}_{4}}$ and ${\gamma }_{{{\text{RaSO}}}_{4}}$ are the activity coefficients of the end-members in the solid solution based on the Thompson-Waldbaum model and assuming a regular mixing model with a Margules interaction parameter, *w*, of 2479 J mol^−1^(cf. Vinograd et al. ^29^). Any solid solution with a stoichiometric supersaturation ${\Omega }_{st}$ > 1 can potentially precipitate while those with a ${\Omega }_{st}$ < 1 will dissolve.

The stoichiometric supersaturation function for all three experiments (line 1, *x* = 0.25 mm) is depicted in Fig. 2c. The maxima of the Ω_st_ at 0.4 < *X*_Ba_ < 0.5 give the thermodynamically most stable solid solution for each experiment. The variation in concentration across the reactor results in variation in the Ω_st_. The evaluation Ω_st_ in the regions, where crystals were observed and monitored, is provided in supplementary note 2.

### Crystallization of (Ba,Ra)SO_4_ solid solutions

**Figure 3:**
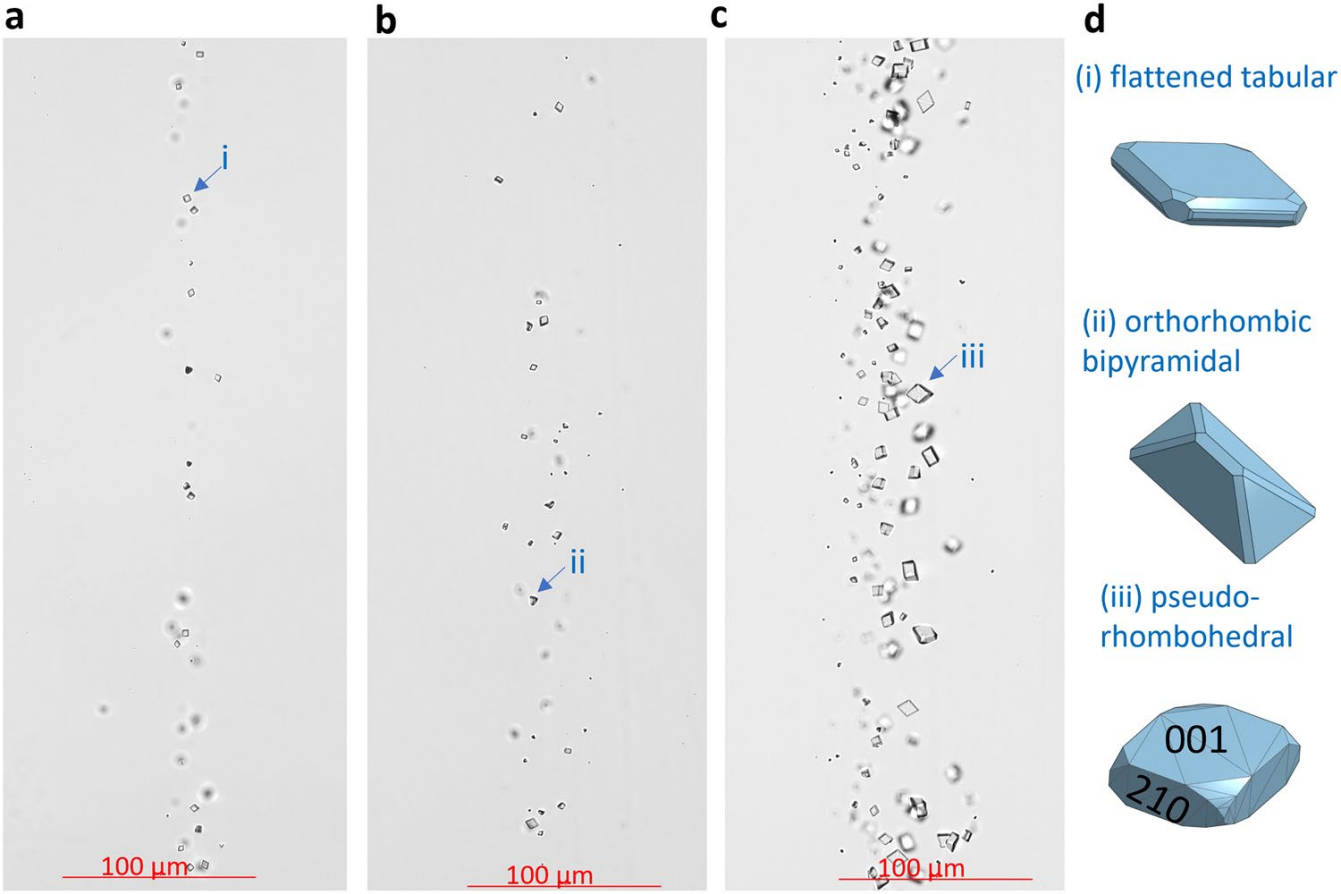
Micrographs of (Ba,Ra)SO_4_ crystals that precipitated in the microfluidic reactor. Sample micrographs from (**a**) experiment A (0.75 mM Na_2_SO_4_) (**b**) experiment B (1 mM Na_2_SO_4_) and (**c**) experiment C (1.5 mM Na_2_SO_4_) with (**d**) the three identified crystals habits that were observed in these experiments. The figures in (**d**) were created using Onshape^68^.

The ingress of the mixed barium chloride/radium bromide solution and sodium sulfate solution into the microfluidic reactor triggered the crystallization of (Ba,Ra)SO_4_, resulting in the formation of euhedral-shaped crystals (Fig. 3). Nucleation started during the seeding procedure at the interface of the mixing solution and the crystallites grew continuously, forming a trail of crystals in the middle of the microfluidic channel (0.245 mm < *x* < 0.255 mm). Fewer crystals of sizes 4.9 ± 0.1 µm were observed for experiment A with 0.75 mM Na_2_SO_4_, while larger crystals merging after 2–3 h were observed for the experiments with higher concentrations of Na_2_SO_4_. Crystal habits were clearly distinguishable, including flattened tabular (Fig. 3d i), orthorhombic bipyramidal (Fig. 3d ii), and pseudo-rhombohedral crystals (Fig. 3d iii), with the latter being the predominant crystal habit observed in all three experiments.

### Determination of solid solution composition

**Figure 4:**
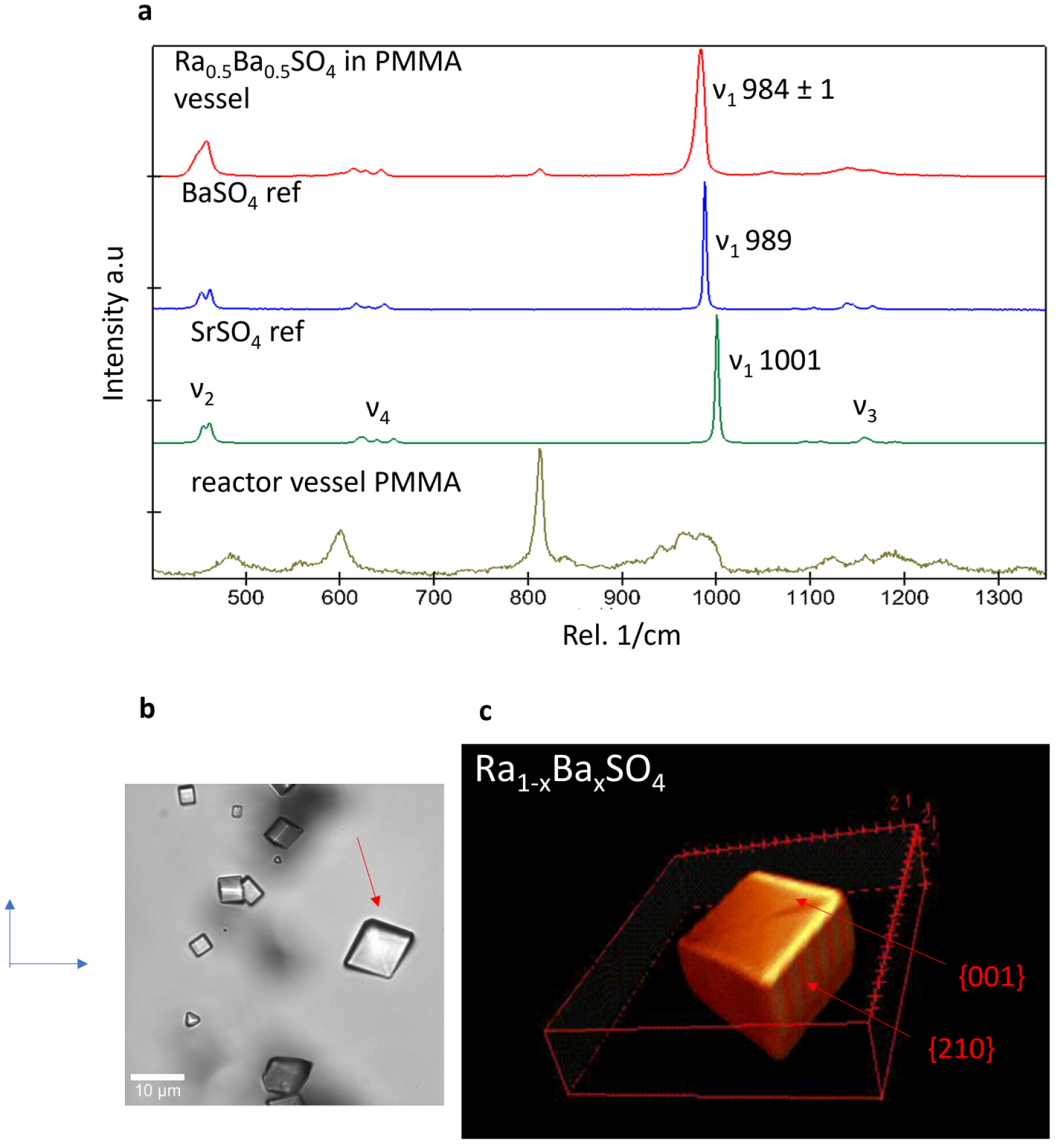
Raman spectroscopic analysis of (Ba,Ra)SO_4_ crystals. (**a**) Raman spectra of a typical crystal that precipitated in the microfluidic reactor and standard spectra of commercial BaSO_4_ and SrSO_4_ and the microfluidic reactor vessel; (**b**) an example of a pseudo rhombohedral crystal of (Ba,Ra)SO_4_ on which further Raman measurements were done to reconstruct its (**c**) 3D geometry.

The Raman spectra for at least fifteen crystals from each experiment were collected to determine the solid solution composition. Figure 4a shows one spectrum as a typical example. In addition, the Raman spectra of synthetic BaSO_4_ (99.99% from Chempur), SrSO_4_ (99.99% from Chempur), and PMMA (reactor material) were collected. These measurements served as standards for the evaluation of our experimental data. The free sulfate ions (SO_4_^–2^) have characteristic ν_1_ and ν_3_ bands corresponding to the symmetric and the anti-symmetric stretching modes, respectively, and ν_2_ and ν_4_ bands corresponding to the bending vibrations. The intense ν_1_(SO_4_) bands for BaSO_4_ and SrSO_4_ are located at 988 cm^−1^ and 1001 cm^−1^, respectively. No Raman data for RaSO_4_ or (Ba,Ra)SO_4_ from literature were available for comparison necessitating further calculations to access this information. The Raman spectra of pure RaSO_4_, BaSO_4_ and SrSO_4_ were determined using DFT calculations, of which a detailed analysis is given in supplementary note 4. Based on these calculations the position of the ν_1_(SO_4_) band would shift by −12 cm^−1^ for RaSO_4_ and + 11 cm^−1^ for SrSO_4_ with respect to that of BaSO_4_. Given that the shift of the ν_1_(SO_4_) for SrSO_4_ is consistent with experimental measurements (Δν =  + 12 cm^−1^) it can be expected that the ν_1_ (SO_4_) position in the Raman spectrum of RaSO_4_ is located at ν_1_ = 977 cm^−1^. According to Vegard’s law, the lattice parameters are correlated to the composition of the solid solution. Lattice parameters (more specifically distance between atom) and composition (more specifically mass of the atom) affect the vibrational frequencies. For the case of solid solution, an increase in one end-member composition usually leads to a continuous change in both the band position and band widths^69^. This means that the linear interpolation of the positions (cm^−1^) of the ν_1_(SO_4_) band maxima is a function of the mole fraction *X*_Ba_ in (Ba,Ra)SO_4_. This was verified experimentally for the case (Ba,Sr)SO_4_ solid solutions despite their non-ideal mixing behavior (c.f. supplementary note in Poonoosamy et al^20^). The measured ν_1_(SO_4_) frequency of all analyzed crystals is distinct, intermediate between those of pure BaSO_4_ and RaSO_4_, and broadened, indicating without any doubt the presence of (Ba,Sr)SO_4_ solid solutions. The ν_1_(SO_4_) band observed at 984 ± 1 cm^−1^ corresponds to a stoichiometric composition of (Ba_0.5_Ra_0.5_)SO_4_ (~ 0.5 × (977 + 989) = 983 cm^−1^).

Stacked 2D Raman images of single pseudo-rhombohedral-shaped crystals (Fig. 4b) were collected to construct their 3D structures (Fig. 4c). Such crystals were observed to grow faster in the *x*–*y* direction than in the *z* direction, e.g., a rhombohedral crystal (Fig. 4c) of 15 ± 1 µm along its diagonal would be around 5 ± 3 µm in depth. These observations suggest that the crystals grow twice as fast along the $\left\{210\right\}$ surfaces (causing growth in the *x*–*y* direction) than on the {001} surface (causing growth in the *z* direction). This observation is frequently reported also for pure barite ^61,62^.

### Computer vision pipeline for the determination of the crystal growth rate

**Figure 5:**
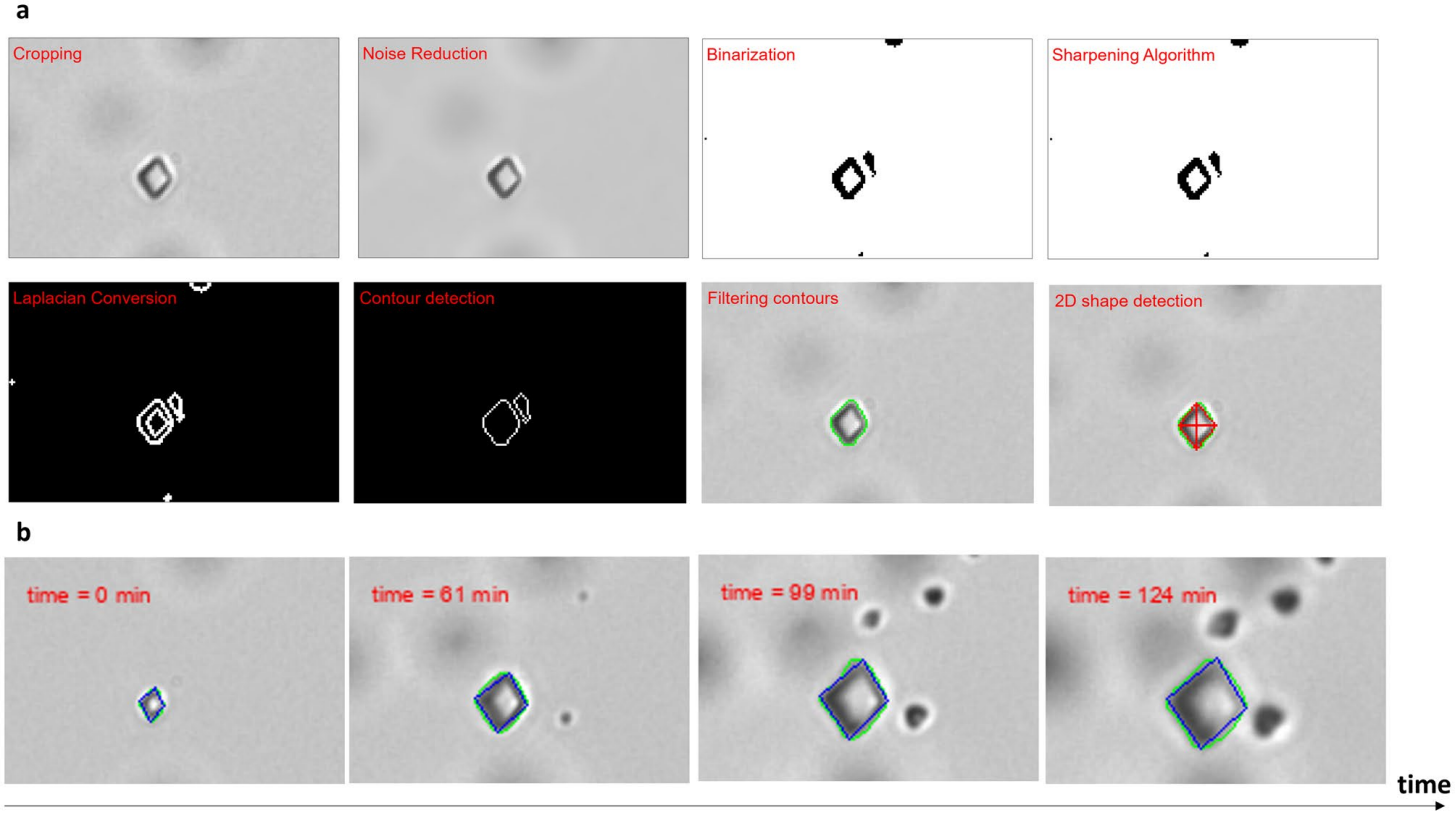
CV pipeline for the identification of crystal habits and determination of crystal growth rates. (**a**) The pre-processing steps for identifying pseudo-rhombohedral crystals (**b**) Tracking the growth of single crystals with time, with the green line capturing the rhomboidal shapes and the blue line approximating the pseudo-rhombohedral 3D crystal habit.

A CV methodology was developed to detect (Ba,Ra)SO_4_ crystals, identify their habits and determine their crystal volume and surface area. We focused our analysis on the pseudo-rhombohedral crystals, the most abundant crystal habit observed. For each experiment, 30 crystals were analyzed. A semi-automated Python tool (supplementary note 3) is employed for the selective analysis of flat crystals from 2D optical microscopy images. The optical microscopy images were pre-processed, which included several steps such as filtering, thresholding, and contour detection, as shown in Fig. 5a, to identify the pseudo-rhombohedral-shaped crystals. To determine volume and consequently the molar amounts of precipitates from 2D images, 3D models based on crystal drawings (Fig. 3d) were integrated into our CV pipeline. The python library Trimesh^70^ (version 3.2.0) was used for 3D modeling of single crystals and accurate volume determination. Surface area calculations were similarly evaluated. Geometrical filters were employed to match 2D shapes of crystals (green line in Fig. 5b) to 3D mathematical shapes (blue line in Fig. 5b) in order to track the 2D surface and volume of the growing crystals with time. A convolutional neural network (CNN) was also developed to identify crystals with a categorized habit. However, due to the currently insufficient amount of data for training, CNN was not able to identify accurately the habits when the crystals were tilted i.e., the crystals were not flat from a 2D perspective.

The volume of single crystals was used to calculate the molar amounts (*n* [mol]) of (Ba_0.5_Ra_0.5_)SO_4_ that precipitated assuming the molar volume of (Ba_0.5_Ra_0.5_)SO_4_ to be proportional to the mole fractions of the end members (c.f. Vinograd et al.^30^) with a value of 53.7 cm^3^ mol^−1^. The rate of precipitation at a given time (*t*), (*R*_*t*_ [mol^−1^ m^2^ s^−1^] normalized to the surface area (*A*_*t*_ [m^2^]) was determined using Eq. 3^53^:

3 $${R}_{t}=\frac{{n}_{t+1}-{n}_{t}}{\Delta t\times {A}_{t}}$$

where Δ*t* is the time lapse between captured images.

**Figure 6:**
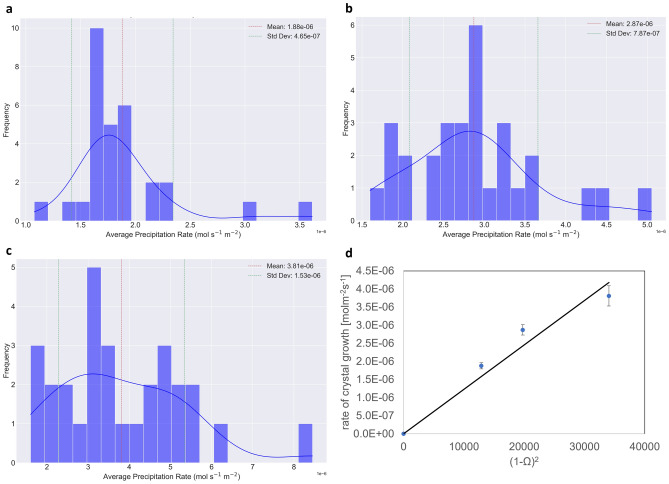
Evaluation of crystal growth rate. Average precipitation rate (*R*_t_) of single crystals for (**a**) experiment A, (**b**) experiment B and (**c**) experiment C. (**d**) Graph of crystal growth rate *R* as a function of supersaturation ratio with respect to Ba_0.5_Ra_0.5_SO_4._ The error associated with the crystal growth rate in (**d**) is the standard error associated with the sampling distribution in (**a**–**c**). The variations in ${\Omega }_{st({{\text{Ba}}}_{0.5}{{\text{Ra}}}_{0.5}{{\text{SO}}}_{4})}$ in the monitored region were included as the uncertainty on ${\Omega }_{st}$ but was insignificant enough to be visible on the graph.

An average rate of precipitation for each crystal was calculated for experiments A-C and reported in the histograms given in Fig. 6. The mean crystal growth rate for each experiment and associated standard deviations were calculated and are given in Fig. 6.

### Determination of the kinetic constant for Ba_0.5_Ra_0.5_SO_4_

The kinetic constant for the crystal growth of B_0.5_Ra_0.5_SO_4_ was calculated based on the measured crystal growth rates from the optical microscopy images. The crystal growth rate normalized to the surface area (*R* [mol m^−2^ s^−1^]) for the precipitated Ba_0.5_Ra_0.5_SO_4_ was assumed to follow a second order reaction like that reported for barite (BaSO_4_). The kinetic constant, *k*, can be calculated using the relationship:

7 $${R}_{({{\text{Ba}}}_{0.5}{{\text{Ra}}}_{0.5}{{\text{SO}}}_{4})}=k({1-{\Omega }_{st({{\text{Ba}}}_{0.5}{{\text{Ra}}}_{0.5}{{\text{SO}}}_{4})})}^{2}$$

where ${\Omega }_{st({{\text{Ba}}}_{0.5}{{\text{Ra}}}_{0.5}{{\text{SO}}}_{4})}$ is the supersaturation ratio with respect to Ba_0.5_Ra_0.5_SO_4_ solid solution (indicated by red stars in Fig. 2c). It is important to note that the variation of ${\Omega }_{st}$ in the regions where crystals were observed and monitored (0.225 mm < *x* < 0.275 mm and −14 mm < *y* < −5 mm) is negligeable on the final evaluation of the results (c.f. Supplementary note 2).

The rate of crystal growth ${R}_{({{\text{Ba}}}_{0.5}{{\text{Ra}}}_{0.5}{{\text{SO}}}_{4})}$ was plotted against ${\Omega }_{st({{\text{Ba}}}_{0.5}{{\text{Ra}}}_{0.5}{{\text{SO}}}_{4})}$ in Fig. 6d, and consequently, *k* was calculated as the slope of the linear equation, yielding (1.23 ± 0.09) × 10^–10^ mol m^−2^ s^−1^. The error associated with the rate is based on the least squares method, which minimizes the sum of the squares of the differences between the observed and predicted values.

Our results were compared with the experimental data provided by Hedström et al.^71^ (see supplementary note S5 for detailed analysis). Based on these experiments, the precipitation rate of (Ba,Ra)SO_4_ normalized to a specific surface area was estimated to be 2.3 × 10^−8^ mol m^−2^ s^−1^. The calculated rate of precipitation associated with the experimental conditions of Hedstroem et al.^71^ using Eq. 7, is 3.2 × 10^−8^ mol m^−2^ s^−1^. These findings demonstrate a significant consistency between our calculated precipitation rate and the experimental data provided by Hedstroem et al.^71^. The crystal growth rate of pure barite is reported as (1.5 ± 0.2) × 10^–11^ mol m^2^ s^−1^ in literature^62^. This is one order of magnitude lower than one of the synthesized Ra-bearing barite in our experiments indicating that the use of default barium growth rate kinetics to describe the (Ra, Ba)SO_4_ solid solution system in the subsurface or groundwater remediation analyses might lead to an overestimation of aqueous radium in solution. By changing the Ba/Ra ratio in the experiments, the radium content in the crystals can be varied. It still needs to be determined whether the kinetic rate of precipitation of (Ba_*x*_Ra_1-*x*_)SO_4_ scales with the Ra content; this will be further investigated in future studies.

## Conclusion

In this work we developed a microfluidic experiment combined with a computer vision tool to determine the kinetic rate of precipitation of (Ba_*x*_Ra_1-*x*_)SO_4_ where radium is present beyond trace amounts—an achievement previously deemed unfeasible. The determined crystal growth rate can be further applied in geochemical calculations for optimizing wastewater treatment and in reactive transport code for a more realistic assessment of radium mobility in the environment for energy extraction or remediation purposes. The unique capabilities of microfluidics in radiochemistry, particularly the ability to conduct multiple experiments in parallel with minimal radionuclide inventories, underscore the potential of this methodology. The developed computer vision tool further enhances experimental throughput, providing robust statistical insights. This methodology will be further developed to conduct experiments at higher temperatures and derive the activation energies. Future work will focus on converting the processing routine from the use-case specific Jupyter notebooks (c.f. code availability) into a python package for generic application. Moreover, we will concentrate on developing a fully automated methodology by implementing CNN into the pipeline.

## Experimental/methods

### Chemicals used

A stock solution with 0.0375 mM of ^226^Ra was prepared from RaBr_2_ (salt, purchased from section Physik Univ. München, Technologisches Labor, München, Germany and manufactured by VG. Khlopin Radium Institute, St Peterburg, Russia in 2003). The Ra activity in solution, from which the concentration is calculated, was quantified via Gamma spectrometry using a N_2_ cooled high purity germanium detector (HPGe, Canberra ɣ-analyst) considering the 186 keV gamma line of ^226^Ra. 10 µL of a 30 mM BaCl_2_ solution (prepared from BaCl_2_·2H_2_O salt > 99% index no. 056-004-00-8 sigma Aldrich), was pipetted in the 5 mL of the Radium solution yielding a mixed solution of 0.061 mM of BaCl_2_ and 0.0375 mM RaBr_2_. Solution of 0.75 mM, 1 mM and 1.5 mM of Na_2_SO_4_ were prepared from Na_2_SO_4_ salt (> 99% Cas no. 7757–82-6).

### Experimental setup

The experimental setup consisted of a microfluidic mixer (Fig. 1) that is connected to syringe pumps (PHD ULTRA™ Syringe Pumps, Havard Apparatus, Massachusetts, United States) and monitored by time-lapse optical microscopy using an inverted Microscope Eclipse Ti2 (NIKON, Tokyo) equipped with a motorized stage and with a 40X objective (CFI Plan Apochromat, NA 0.95, Nikon, Tokyo). The microfluidic mixer made out of polymethylmethacrylate (PMMA), with dimensions shown in Fig. 1, consists of two inlets and one outlet. The two inlets (1 and 2) were each connected to 500 µL glass syringes, dispensing the mixed solution of BaCl_2_ and RaBr_2_ and Na_2_SO_4_, respectively (Fig. 1). The outlet was linked to an effluent vessel. A PMMA reactor was chosen over the classically used polydimethylsiloxane (PDMS) because it is gas-tight, making it safer for handling radionuclides. The inlets and outlets (mini luer) were connected with tubings (Tygon AAD04103, ID 0.51 mm, OD 1.53 mm, Saint-Gobain Performance Plastics, Akron, OH, USA) via mini luer connectors (chipshop GmbH, Jena, Germany). The microfluidic reactor was initially filled with deionized water, followed by the injection of the reacting solutions at 1 µL min^−1^ for 15 min. The syringe pump was stopped for 15 min to enable nucleation in the microfluidic channel (seeding step). After these 15 min, the pump was switched on again and reacting solutions were injected at 1 µL min^−1^ and monitored by optical microscopy for 3 h. Three experiments were conducted with varying concentrations of Na_2_SO_4_ of 0.75 mM, 1 mM and 1.5 mM and were labeled A, B and C, respectively. The experiment was conducted at ambient temperature (21 °C) and pressure. Micrographs of 439 µm × 439 µm were collected using a high-resolution camera from Zyla (sCMOS, Andor, Belfast) in DIC mode over 11 to 15 areas of the 10 mm monitored region (Fig. 1) at regular intervals of 5 min.

### Transport in microfluidic mixer

The flow field in the microfluidic mixer was simulated with computational fluid dynamics using the software COMSOL Multiphysics 6.0 (COMSOL AB, Stockholm, Sweden see supplementary note 1). The initial saturation ratio with respect to the complete solid solution series was calculated following the steps described in Poonoosamy et al.^72^. This process involved an initial evaluation of the flow and concentration fields using COMSOL Multiphysics. Subsequently, the geochemical calculations of aqueous speciation were performed using GEMS selektor^65^. The resulting activities of aqueous species from the geochemical solver are used for the computation of the supersaturation ratio function given in Eq. 2.

### Raman measurements

Raman measurements and 3D tomographs were conducted using a Witec alpha300 Ri Inverted Confocal Raman Microscope with a Nikon 100 × oil immersion objective, having a numerical aperture (NA) of 1.4, a working distance of 0.13 mm, and a cover glass correction. The instrument is equipped with a 70 mW Nd:YAG laser (*λ* = 532 nm) and a thermoelectrically cooled charge-coupled device (CCD). The laser power was set to 20 mW and a grating with 1800 grooves per mm was chosen. With this setup, the spectral resolution was 2 cm^−1^. The theoretical, diffraction-limited lateral and axial resolutions of the Raman measurements at the sample surface were ~ 464 nm and ~ 629 µm considering Eqs. (3) and (4) in Everall^73^, and the refraction index of the immersion medium (*n* = 1.55). Raman spectra of at least 15 crystals per experiment were collected with a measuring time of 20 s in the wavenumber range from 300 cm^−1^ to 1400 cm^−1^. In addition, stacked Raman images of single crystals were collected with a 500 nm step size in the *x* and *y* direction over a depth of 10 μm with a 1 µm step size. Raman intensities were recorded for 0.1 s in the wavenumber range from 300 cm^−1^ to 1400 cm^−1^. These images were used to reconstruct the 3D geometry of the single crystals, enabling the determination of their volume. Raman image stacks were visualized with the ImageJ 3D Viewer (version 4.0.2).

### DFT calculation

The synthetic Raman spectra of BaSO_4_, RaSO_4_ and SrSO_4_ compounds were computed with the plane-wave Quantum-ESPRESSO package^74^, using density functional perturbation theory. We applied the norm-conserving pseudopotentials, the kinetic energy cutoff of 200 Ry and the LDA (for Raman intensities) and PBEsol (for vibrational frequencies) exchange–correlation functionals^75^. The compounds were modeled with four formula units supercells that contained 24 atoms and 2 × 2 × 2 Monkhorst–Pack k-point grid for Brillouin zone^76^ sampling. Details of these calculations and analyses are given in supplementary note 2.

### Computer vison pipeline

The development of the CV methodology for (Ba,Ra)SO_4_ crystal analysis involved some carefully designed steps that allowed us to extract physical details and growth rates from 2D optical microscopy images. This included a semi-automated image-processing Python methodology that allows for bordering of selected regions of interest from the available TIFF input images. This is essential as it ensures the exclusion of crystals that are not flat from a 2D perspective. Analyzing non-flat crystals can potentially result in inaccurately identifying an enlarged perimeter, leading to inaccurate calculations of volumes and precipitation rates. It is important to mention that the selection process is conducted on the final image of each experiment to ensure the capture of the necessary coordinates. These coordinates are then saved and utilized by the CV tool to trace the growth of crystals throughout the entire duration of the experiment.

The first step of the main CV code, the pre-processing step, employs various Python libraries such as OpenCV^77^ (version 4.6.0.66) for image processing, Pandas^78^ (version 1.3.3) for data manipulation, and Matplotlib^79^ (version 3.4.3) for graphical representation. This step involves applying bilateral filtering^80,81^ to reduce noise around the border of the crystal while preserving edges, adaptive thresholding^82,83^ to segment the crystals from their background, and contour detection^84^ to identify the crystal shapes. The contours are then filtered further to exclude small artifacts and nested contours in order to ensure that only the valid crystal shape is considered. Geometrical filters are then applied to detect the 2D shape and match it with its corresponding 3D morphology. For example, detected rhomboidal shapes are matched with pseudo-rhombohedral 3D morphologies.

Following the image pre-processing step, crystallographic 3D morphological models were drawn using the CAD software *onshape*^68^, and were designed based on drawings from the (Atlas der Krystallformen) textbook^85^. These 3D models are then integrated into the proposed CV tool to calculate the volume of the crystals being analyzed. As for the growth rate of the unseen dimension from the 2D perspective, the depth is determined based on the relevant available research work and experimentally determined 3D structures. For example, for the pseudo-rhombohedral morphology, the growth rate of the {210} surface is approximately twice as fast as that of the {001} surface^61,62^. In the main code, the 3D modeling is utilized using the Trimesh library^70^ (version 3.2.0), with the volume of the crystal being calculated based on the scaled dimensions derived from the two-dimensional analysis. The scaling is done in a way that ensures the proportions are accurately maintained, and that the volumes are calculated by scaling a 3D mesh model to accurately match the rate of growth of the visible dimensions of the crystal that were observed by the CV tool during the experiment. It is also important to mention that the maximum depth was manually set to be 10 µm as it is the actual depth of the experimental microfluidic chambers. This methodology allowed us to predict the depth of the observed crystals accurately according to the representation of the crystal’s 3D morphology. Additionally, it was possible to calculate the surface area of the scaled mesh, providing additional data needed for the calculation of the precipitation rate. The precipitation rate is calculated using Eq. 3. The surface area is estimated by updating its value for each time interval.

### Supplementary Information

Supplementary Information.

## Data Availability

A detailed description of the numerical methods fluid dynamics simulation, computer vision pipeline and DFT calculations is given in the supplementary notes 1, 2 and 3 respectively. The datasets for graphs in Figs. 2 and 6 including, Raman spectra (in txt format) as well as videos of crystal growth from experiment 1–3 can be found on a public repository https://b2share.eudat.eu/records/f46a0f28a48644d5bc15d6296b88120a. Any other datasets, e.g., images or COMSOL simulation files, generated during the current study are also available from the corresponding author upon reasonable request.

## Code availability

The Python code for the CV methodology is available as Jupyter notebooks, which are deposited in a public Git repository on GitHub, reachable via the following URL: https://github.com/FZJ-Reactive-Transport-Group/CV-tool-for-crystals-analysis.
