# Supplementary material for: A radiochemical lab-on-a-chip paired with computer vision to unlock the crystallization kinetics of (Ba,Ra)SO4
Source: Sci Rep. 2024 Apr 25;14:9502. doi: 10.1038/s41598-024-59888-6 (PMC11045812; doi:10.1038/s41598-024-59888-6)
Supplement: Supplementary file 1 — Supplementary Information. [file 41598_2024_59888_MOESM1_ESM.docx]

**Supplementary Information**

**A radiochemical lab-on-a-chip paired with computer vision to unlock the crystallization kinetics of (Ba,Ra)SO_4_**

Jenna Poonoosamy^1^*, Alexander Kaspor^1^, Christian Schreinemachers^1^, Dirk Bosbach^1^, Oskar Cheong^2,3,4^, Piotr M Kowalski^2,3^ Abdulmonem Obaied^1^

1 Institute of Energy and Climate Research (IEK-6): Nuclear Waste Management, Forschungszentrum Jülich GmbH, 52425 Jülich, Germany

2 Institute of Energy and Climate Research (IEK-13): Theory and Computation of Energy Materials, Forschungszentrum Jülich GmbH, 52425 Jülich, Germany

3 JARA Energy & Center for Simulation and Data Science (CSD), 52425 Jülich, Germany

4 Chair of Theory and Computation of Energy Materials, Faculty of Georesources and Materials Engineering, RWTH Aachen University, Intzestrasse 5, 52072 Aachen, Germany

Corresponding author: Jenna Poonoosamy

email: [j.poonoosamy@fz-juelich.de](mailto:j.poonoosamy@fz-juelich.de)

Tel number:  +49 2461 61-6468

## Supplementary Note 1

**Velocity fields and aqueous solute distributions in the reactor**

The fluid flow and transport of solutes in the complete microfluidic mixer were modelled using a 3D finite element approach, in order to characterize the flow field and aqueous concentrations of solutes in the microfluidic reactor. For these scoping calculations, we assumed steady state and ignored the dissociation of the salts in charged ions as well as chemical reactions.

The [velocity field](https://www.sciencedirect.com/topics/earth-and-planetary-sciences/velocity-distribution) in the reactor was calculated using the Navier-Stokes equation [(S1)](https://www.sciencedirect.com/science/article/pii/S0009254119303651#fo0020) for laminar flow of an isothermal, incompressible and [Newtonian fluid](https://www.sciencedirect.com/topics/earth-and-planetary-sciences/newtonian-fluid),

$\rho\left( \boldsymbol{u}\cdot\nabla\right)\boldsymbol{u}=-\nabla p+\mu\nabla^{2}\boldsymbol{u}; \nabla\cdot\boldsymbol{u}=0$ (S1)

where ρ is the density (kg m^-3^), u the fluid velocity (m s^-1^), p the fluid pressure (Pa), and µ the fluid’s dynamic viscosity (Pa s).

The concentrations of solutes in the microfluidic reactor are then computed by solving the diffusion and [advection](https://www.sciencedirect.com/topics/earth-and-planetary-sciences/advection) equation (S2) where C_i_ (i = 1, 2, 3…m) denotes the molar concentration (mol m^−3^) of the ith of m components in the system; u is the velocity (m s^−1^) of the fluid flow, and *D*_i_ is the [diffusion coefficient](https://www.sciencedirect.com/topics/earth-and-planetary-sciences/diffusion-coefficient) of component i.

$\nabla\cdot(\boldsymbol{u}C_{i})-\nabla\cdot\left( D_{i}{\nabla C}_{i} \right)=0$ (S2)

The equations were solved for the given boundary conditions using the computational fluid dynamics software COMSOL Multiphysics 6 (COMSOL AB, Stockholm, Sweden). The flow in the microfluidic reactor was modelled considering a 2D geometry (Figure S1). No-slip condition was applied at the walls of the reactor and a velocity of 1.67×10^-2^ m s^-1^ was specified at each of the two inlet channels. A reference pressure of 0 Pa was specified at the outlet. For the fluid properties, a density of 1000 kg m^−3^ and a viscosity of 8.9 × 10^−4^ Pa s (i.e., referring to pure water) were assumed.


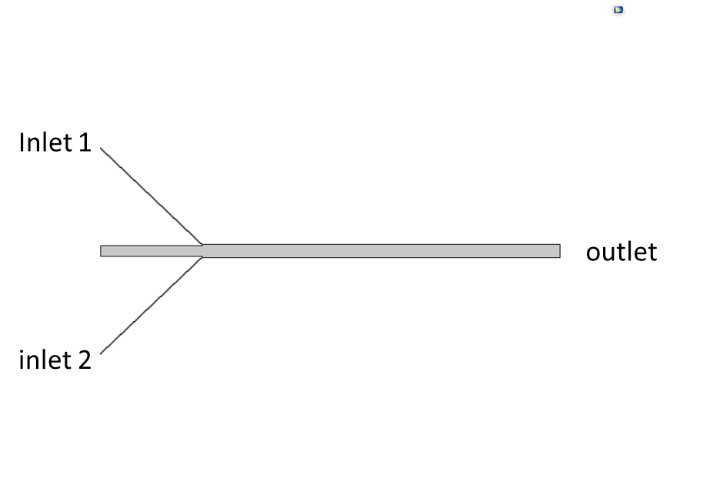


**Figure S1** 2D geometry for COMSOL simulation

*D*_i_ is the diffusion-dispersion coefficient of component i set to 1.23×10^-9^ m^2^ s^-1^ for Na_2_SO_4_ (average for Na^+^ (1.33×10^-9^ m^2^s^-1^) and SO_4_^2-^ (1.07×10^-9^ m^2^ s^-1^)), 1.39×10^-9^ m^2^ s^-1^ for BaCl_2_ (average for Ba^2+^ (8.47×10^-9^ m^2^ s^-1^) and Cl^-^ (2.03×10^-9^ m^2^ s^-1^)) and 1.44×10^-9^ m^2^ s^-1^ for RaBr_2_ (average for Ra^2+^ (0.889×10^-9^ m^2^ s^-1^) and Br^-^ (2.08×10^-9^ m^2^ s^-1^)).

The diffusion coefficients, $D_{salt}$, were calculated as follows^1^:

| $D_{salt}=\frac{(z^{+}+\left\vert z^{-} \right\vert)D_{i}^{+}D_{i}^{-}}{z^{+}D_{i}^{+}+\left\vert z^{-} \right\vert D_{i}^{-}}$ | (S3) |
| --- | --- |

where $z$ is the charge of the ion (e.g., $z_{i}$ = -2 for SO_4_^2-^), and $D_{i}$ is the ion diffusion coefficient. The potential effects of Coulombic interactions on mass transfer of the different ions were ignored in the modelling approach.

This step enables the determination of the time when steady state is reached and of the initial concentration gradients in the system.

## Supplementary Note 2

**The stoichiometric supersaturation with respect to Ba_0.5_Sr_0.5_SO_4_ in the reactor**


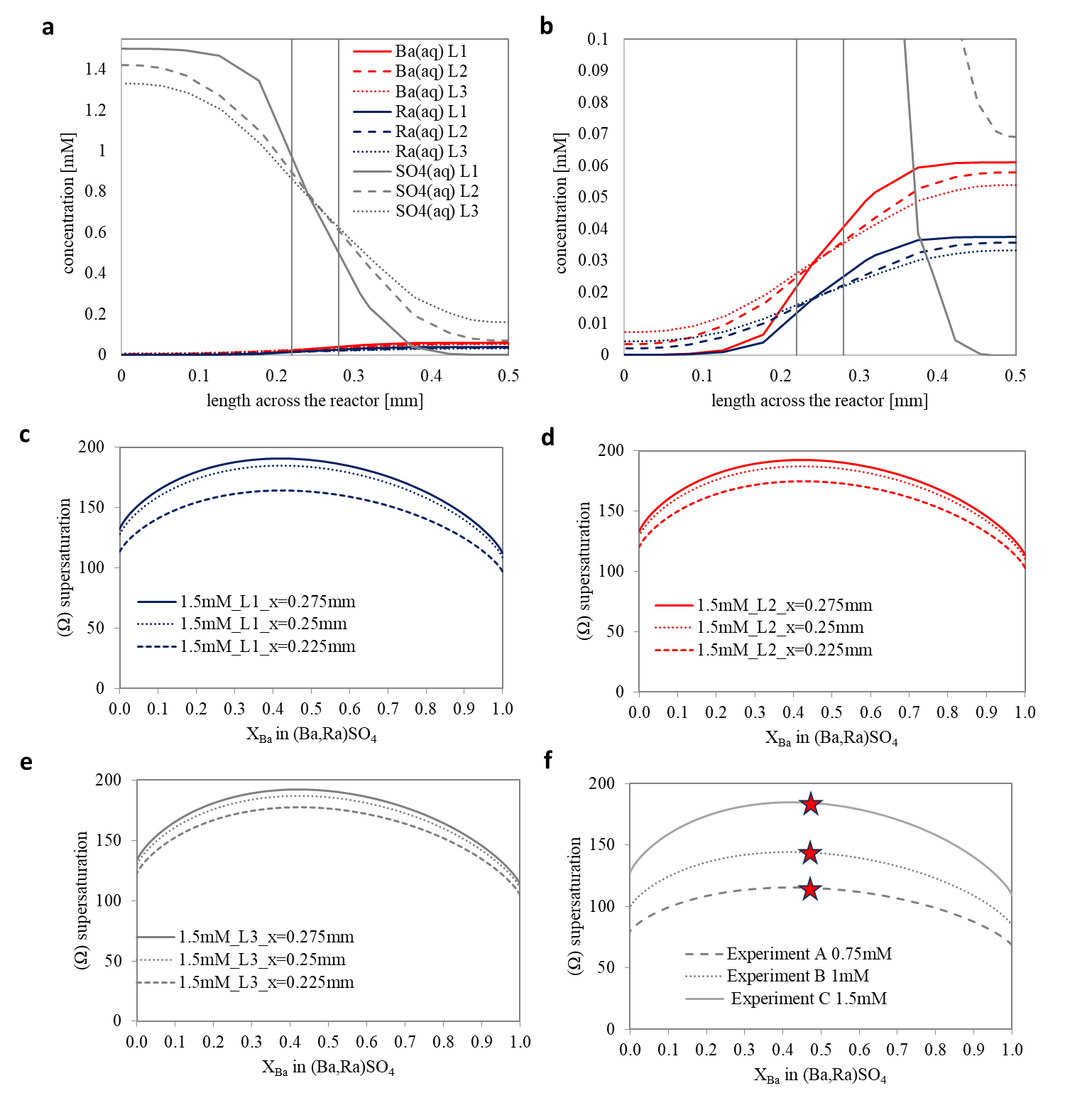


**Figure S2.** Evaluation of the solution chemistry and supersaturation function Ωst along the mixing zone. (a) The concentration of solutes across line 1-3 (c.f. Figure 1) for experiment C with (b) a change in the y-axis for a better visualization of Ba(aq) and Ra(aq). The Ωst on three points along (c) line 1, (d) line 1 and (e) line 3 and (f) the comparison of the Ωst for experiment A, B and C along line 1 at x = 0.25 mm with the red stars indicating the thermodynamically most stable solid solution composition based on the aqueous solution chemistry.

The stoichiometric supersaturation functions were computed on 3 points (*x* = 0.225 mm, *x* = 0.25 mm and *x* = 0.275 mm) on lines 1-3. The results for experiment C (experiment with 1.5 mM Na_2_SO_4_) are presented as an example in Figure S2c-e. The $Ω_{st}$ varies across the *x*-axis of the reactor (Figure S2c-e), with a rather constant Ω_st_ value along the middle of the reactor, i.e., along *x* = 0.25 mm, with a slightly higher Ω_st_ observed towards the barium-radium side (*x* = 0.275 mm) and a lower Ω_st_ toward the sulfate side (*x* = 0.225 mm). This is explained by the higher concentrations of sulfate used in the experiments. The slightly different ion diffusion coefficient plays a negligible role here in the distribution of ions across the mixing zone in comparison to the concentration. The stoichiometric supersaturation function for all three experiments (line 1, *x* = 0.25 mm) is depicted in Figure S2f. The maxima of the Ω_st_ at 0.4 < *X*_Ba_ < 0.5 give the thermodynamically most stable solid solution for each experiment.

## Supplementary Note 3

**Description of the main Computer-Vision (CV) techniques used for analyzing the 2D images.**

In this section we detailed the different pre-processing techniques to evaluate the crytal growth rate from the 2D images.

**Bilateral filtering:**

This approach stands out in the image processing domain as a crucial method that excels in reducing the noise occurring in images while simultaneously preserving the clarity of shape edges with precision. This non-linear method updates the value of each pixel by calculating an average that takes into account the values of nearby pixels. This is done while keeping focus on those pixels that are close in space and similar in intensity. These average are calculated using Gaussian functions, which assign more importance to neighboring pixels that are both spatially close and similar in intensity, which preserves the edges' clarity.

Mathematically, the bilateral filter B(I) for an image I at pixel x is defined as:

$B\left( I \right)_{x}=\frac{1}{W_{p}}\sum_{x_{i}\in\Omega} I_{x_{i}}\cdot f\left( |x-x_{i}| \right)\cdot g\left( \left| I_{x}-I_{x_{i}} \right| \right)$ (S4)

Where $B\left( I \right)_{x}$ represents the intensity of the output image at pixel x, $I_{x_{i}}$ denotes the intensity of the input image at a neighboring pixel *x*_i_, Ω is the neighborhood around pixel x, f is a Gaussian function measuring the spatial distance between *x* and *x*_i_, g is another Gaussian function measuring the intensity difference between the pixels and $W_{p}$ is a normalization term ensuring that the weights sum up to 1

This dual capability of smoothing noise while preserving edge positions, the bilateral filter is a superior choice compared to other filters, like Gaussian filters, which might blur edges while removing noise. Its applications span across computer vision, medical imaging, and computer graphics, showcasing its versatility and effectiveness in complex image processing tasks.

**Adaptive thresholding**

This technique is frequently employed to separate objects from their surroundings, a task that gains critical importance in situations where the lighting across an image is not uniform. Diverging from the approach of global thresholding, which assigns a uniform threshold level to the entire image, this adaptive method calculates distinct threshold values for localized areas. This tailored computation enhances the method's sensitivity to the diverse lighting conditions within different segments of the image.

$T\left( x,y \right)=mean\left( R\left( x,y \right) \right)-C$ (S5)

where *R*(x,y) represents a local region around the pixel (x,y), mean(*R*(x,y)) calculates the mean pixel value within this region, and *C* is a constant subtracted to tailor the thresholding. The pixel (x,y) is then classified as an object if its intensity is less than *T*(x,y) and as background otherwise ^2^.

This method proves to be especially beneficial when dealing with images that exhibit fluctuating light conditions. It adeptly modifies the threshold in response to the image's local nuances, which results in segmentation that is both more precise and steadfast. The selection of the region size and the constant C is pivotal, as these factors govern the method's sensitivity and overall success. If the region chosen is too wide, it could undermine the adaptability of the approach. Conversely, a region that is too diminutive might intensify the noise^3^ .

**2D filtering**

The filter2D function is considered an essential image processing technique, which offers enough flexibility for convolving an image using a given kernel. This function’s effectiveness in enhancing image quality and extracting information is well-documented in the literature ^2,4^. It is mainly used to execute an array of operations, such as blurring, sharpening, embossing, edge detection, and more. The operation is mathematically defined by the convolution of the image I with a kernel K, which can be represented as:

$I^{'}\left( x,y \right)=\sum_{i=-k}^{k} \sum_{j=-k}^{k} I\left( x-i,y-j \right)\cdot K\left( i,j \right)$ (S6)

where $I^{'}\left( x,y \right)$ is the transformed pixel value, $\left( x-i,y-j \right)$ are the original pixel values of the image, and $K\left( i,j \right)$ are the coefficients of the kernel matrix. The kernel is a small matrix used to apply certain effects such as blurring or sharpening. The size of the kernel affects the extent of the convolution, with larger kernels producing more pronounced effects. In practice, the kernel is slid over the image, and at each location, the sum of the product of the kernel coefficients with the corresponding image pixel values is computed. This sum then replaces the value of the central pixel of the output image at the current location.

**Laplacian filtering**

This method utilizes a second-order derivative operator for edge detection in image processing^5^ . The operation applies a Gaussian smoothing to reduce noise followed by the Laplacian filter. It operates by approximating the Laplace of Gaussian (LoG), which measures how the intensity rate of images change at each pixel location. The mathematical representation of the Laplacian operator is given by the sum of the second derivatives of the image intensity *I* with respect to the spatial coordinates x and y:

$L\left( x,y \right)=\frac{\partial^{2}I}{\partial x^{2}}+\frac{\partial^{2}I}{\partial y^{2}}$ (S7)

## Supplementary Note 4

**Raman spectra of BaSO_4_, RaSO_4_ and SrSO_4_**

The synthetic Raman spectra of BaSO_4_, RaSO_4_ and SrSO_4_ compounds were computed with the plane-wave Quantum-ESPRESSO package, using density functional perturbation theory as implemented in Phonon tool^6^. We applied the norm-conserving pseudopotential, the kinetic energy cutoff of 200 Ry and the LDA(for Raman intensities) and PBEsol (for vibrational frequencies) exchange-correlation functionals^7^. The compounds were modeled with four formula units supercells that contained 24 atoms and 2x2x2 Monkhorst–Pack k-points grid for sampling of Brillouin zone^8^. The structures were pre-optimized, and the resulting volumes are provided in Table S1.

In Figure S3 we collected all the computed Raman spectra of BaSO_4_, RaSO_4_ and SrSO_4_. These spectra are consistent with experimental datasets, with the pronounced ν_1_ band associated with vibrations of SO_4_ tetrahedra around 950 cm^-1^. The position of the ν_1_ band for each compound is given in Table S1. The computed spectrum is shifted by ~ 40 cm^-1^, but such a systematic offset is a regular feature of DFT calculations^9^.

Table S1 compares the Raman peaks and cell volumes of all three compounds computed with DFT. The cell volumes are in good agreement with experimental results. Based on these calculations (PBEsol) the position the ν_1_(SO_4_) band would shift by -12 cm^-1^ for RaSO_4_ and +11 cm^-1^ for SrSO_4_ w.r.t that of BaSO_4_. Given that the shift of the ν_1_ (SO_4_) for SrSO_4_ is consistent with experimental measurements (Δν_1_ = +12 cm^-1^) it can be expected that the ν_1_ (SO_4_) position in the Raman spectrum of RaSO_4_ is located at ν_1_ = 977 cm ^-1^. We therefore consider in our analysis that the ν_1_ band of RaSO_4_ is located at 977 cm^-1^.

**Table S1** Comparison of cell volumes and main Raman peak frequency ν_1_ (SO_4_) of BaSO_4_, RaSO_4_ and SrSO_4_

| Compound | cell volume [A^3^] | | Wavenumber of *ν*1 band [cm^-1^] | | calculated deviation from BaSO_4_ ν_1_ [cm^-1^] | |
| --- | --- | --- | --- | --- | --- | --- |
|  | PBEsol | experimental | PBEsol | experimental | PBEsol | experimental |
| BaSO_4_ | 346 | 343 | 945 | 989 | - | - |
| RaSO_4_ | 377 | 364 | 933 | - | -12 | - |
| SrSO_4_ | 307 | 308 | 956 | 1001 | 11 | 12 |


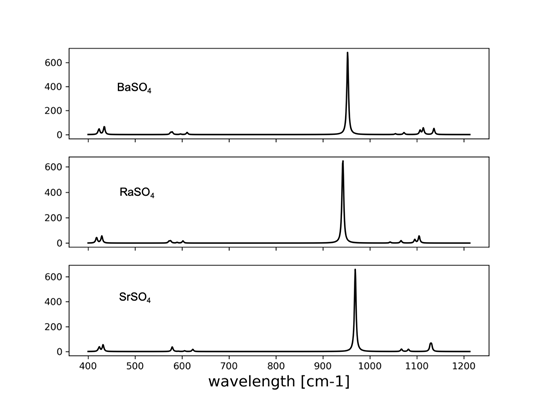


**Figure S3:** Computed Raman spectra for BaSO_4_, RaSO_4_ and SrSO_4_ compounds.

## Supplementary Note 5

**Comparison of our results with Hedström et al. et al^10^**

A direct comparison of the kinetic rate reported in this work and Hedström et al.^10^ is not possible, as the rate is not given as a function of supersaturation ratio and is not normalized to the surface area as in the current work and commonly reported in the geoscience community (e.g. <https://pubs.usgs.gov/of/2004/1068/>). Moreover, the absence of chemical characterization of the precipitates by standard tools like XRD/SEM or Raman makes it difficult to determine if a solid solution of the same chemical composition was synthesized in Hedström et al.^10^. The data needed for further processing.

Using Equation 1 and coefficients from Table 1 by Hedström et al.^10^, the kinetic constant at 293.15K is calculated to be 4.6 x 10 ^-6^ mol s^-1^. Assuming a specific surface area of 0.5 m^2^ g^-1^ (BET surface area reported for barite from Bosbach et al.^11^), re-evaluated to 138.9  m^2^ mol^-1^ for Ba_0.5_Ra_0.5_SO_4_, we estimated a precipitation rate normalized to the surface area as 2.3  x 10^-8^ mol  m^-2^  s^-1^.

The kinetic rate in our study, being a function of supersaturation, requires re-evaluation for comparison. We computed the geochemical speciation of the solution and the stoichiometric saturation function, Ωst, based on the data provided in Hedström et al.^10^ (RaCl_2_ 7  x 10 ^-7^ M; Ba(NO_3_) 7  x 10 ^-7^ M; Na_2_SO_4_ 2  x 10 ^-2^ M; pH 3; temperature 293.15K). The Ωst of the solution is 17.21 with respect to Ba_0.5_Ra_0.5_SO_4_. Utilizing Equation 7 from the main manuscript, the rate associated with the solution composition of Hedström et al. can be computed as 1.23 × 10 ^-10^ × (1-17.2)^2^ = 3.2  × 10^-8^ mol m ^-2^ s^-1^.

These findings demonstrate a significant consistency between our calculated precipitation rate and the experimental data provided by Hedstroem et al.^10^.

**References**

1 Lasaga, A. C. The treatment of multi-component diffusion and ion pairs in diagenetic fluxes. *Am. J. Sci.* **279**, 324-346 (1979).

2 Gonzalez, R. & Faisal, Z. *Digital Image Processing Second Edition*. (2019).

3 Sezgin, M. & Sankur, B. Survey over image thresholding techniques and quantitative performance evaluation. *J Electron Imaging* **13**, 146-168, doi:10.1117/1.1631315 (2004).

4 Jain, A. K. *Fundamentals of digital image processing*. (Prentice-Hall, Inc., 1989).

5 Marr, D., Hildreth, E. & Brenner, S. Theory of edge detection. *Proceedings of the Royal Society of London. Series B. Biological Sciences* **207**, 187-217, doi:doi:10.1098/rspb.1980.0020 (1980).

6 Giannozzi, P. *et al.* QUANTUM ESPRESSO: a modular and open-source software project for quantum simulations of materials. *J. Condens. Matter Phys.* **21**, 395502, doi:10.1088/0953-8984/21/39/395502 (2009).

7 Perdew, J. P. *et al.* Restoring the Density-Gradient Expansion for Exchange in Solids and Surfaces. *Phys. Rev. Lett.* **100**, 136406, doi:10.1103/PhysRevLett.100.136406 (2008).

8 Monkhorst, H. J. & Pack, J. D. Special points for Brillouin-zone integrations. *Phys. Rev. B* **13**, 5188-5192, doi:10.1103/PhysRevB.13.5188 (1976).

9 Jahn, S. & Kowalski, P. M. Theoretical Approaches to Structure and Spectroscopy of Earth Materials. *Rev. Mineral. Geochem.* **78**, 691-743, doi:10.2138/rmg.2014.78.17 (2014).

10 Hedström, H., Ramebäck, H. & Ekberg, C. A study of the Arrhenius behavior of the co-precipitation of radium, barium and strontium sulfate. *J. Radioanal. Nucl. Chem.* **298**, 847-852, doi:10.1007/s10967-013-2431-0 (2013).

11 Bosbach, D. in *Water-rock interactions, ore deposits, and environmental geochemistry: A tribute to David A. Crerar* Vol. 7 (ed Roland; Wood Hellmann, Scott A.) 97-110 (Geochemical Society special publication, 2002).
